# Coagulation and platelet function in cold‐stored whole blood on missions in a helicopter emergency service

**DOI:** 10.1111/aas.14568

**Published:** 2025-01-19

**Authors:** José‐Gabriel Sato Folatre, Agneta Wikman, Vladimir Radulovic, Göran Sandström, Gabriel Skallsjö, Per Arnell, Sven‐Erik Ricksten, Birgitta Romlin

**Affiliations:** ^1^ Helicopter Emergency Medical Service Region Västra Götaland Kungälv Sweden; ^2^ Department of Anaesthesiology and Intensive Care, Institute of Clinical Sciences, Sahlgrenska Academy University of Gothenburg Gothenburg Sweden; ^3^ Centre for Haematology and Regenerative Medicine (HERM) Karolinska University Hospital Stockholm Sweden; ^4^ Department of Clinical Science, Intervention and Technology (CLINTEC) Karolinska Institute Stockholm Sweden; ^5^ Department of Haematology and Coagulation Disorders Sahlgrenska University Hospital Gothenburg Sweden; ^6^ Department of War Studies Swedish Defence University Stockholm Sweden; ^7^ Department of Paediatric Anaesthesia and Intensive Care, Queen Silvia Children's Hospital, Sahlgrenska Academy University of Gothenburg Gothenburg Sweden

**Keywords:** coagulation, critical emergency medicine, helicopter emergency medical service, platelet function, prehospital emergency services, thromboelastography, whole blood

## Abstract

**Background:**

Haemorrhage is a leading cause of morbidity and mortality in trauma, and prehospital transfusion of blood products is often necessary. Whole blood has been proposed to be the best alternative, but it is unclear whether, and how, storage and transport of the blood in a helicopter affects the blood units. We investigated the coagulation capacity and platelet function in whole blood at different time points during helicopter missions.

**Methods:**

Twenty units of low‐titre group O RhD negative whole blood were collected from healthy volunteers and analysed before, during and after transport in a helicopter. Coagulation and platelet function, as measured by thromboelastography, and blood samples for pH, electrolytes, glucose and lactate were assessed at baseline and 24, 72 and 168 h after storage in the helicopter. Plasma concentrations of coagulation factors and haemoglobin and blood counts were measured at baseline and after 168 h.

**Results:**

Plasma concentrations of coagulation factors and haemoglobin did not change during storage and transport. Platelet counts decreased from a baseline mean of 172 ± 29 × 10^9^/L to a mean of 120 ± 28 × 10^9^/L after 168 h, and platelet function worsened slightly but significantly by 8%–9% during storage and transport. pH and glucose decreased while potassium and lactate levels increased after 168 h compared with baseline.

**Conclusion:**

Storage and transport of whole‐blood units in a rescue helicopter, for up to 168 h, had a slight impact on the blood quality. Storage of whole blood on board of the helicopter holds up to European standard, measured as temperature and haemolysis.

**Editorial Comment**

Prehospital transfusion of whole blood in trauma care is increasingly recognised, yet concerns about the effects of storage and transport on blood quality remain. This study provides evidence that whole blood transported in rescue helicopters maintains acceptable coagulation capacity and platelet function although platelet counts are decreased over 168 h, supporting the use of whole blood in emergency trauma scenarios.

## 1 INTRODUCTION

Haemorrhage is a leading cause of morbidity in trauma care and one of the greatest causes of mortality, contributing to one in 10 deaths worldwide.^1^ In Sweden, traumatic injuries rank as the top cause of death in individuals under 40 years of age,^2^ with the majority of deaths occurring outside hospital. There is a controversy on the use of blood products for resuscitation in haemorrhagic trauma. Current guidelines^3^ do not have a clear recommendation regarding the use of prehospital blood products. The pan‐European, multidisciplinary Task Force for Advanced Bleeding Care in Trauma still recommend 0.9% sodium chloride or a balanced crystalloid solution as initial fluid resuscitation in the hypotensive bleeding patient.^3^ The use of blood component therapy, involving separating whole blood into components and then reconstituting the components with storage solutions, adding up to three times the original volume, may cause dilutional coagulopathy and impaired patient outcome.^4^, ^5^ Some retrospective data support the use of prehospital whole blood.^6^ Viscoelastic haemostatic essays as guidance in trauma resuscitation^7^ have been increasingly used and are recommended in the guidelines.^3^ However, data from recent studies^8^, ^9^ on their beneficial effect, versus conventional coagulation tests, on outcomes such as morbidity, mortality and the use of blood products are conflicting.

Whether whole blood or blood components are used in an emergency medical service, little but some data exist^10^ regarding the quality of cold‐stored whole blood or blood products, regarding platelet function, coagulation capacity, pH, plasma levels of glucose and lactate, and how these variables are affected over time before use in traumatic haemorrhagic shock.

In 2019, the ambulance helicopter in the West Region of Sweden (Västra Götalandsregionen [VGR]) introduced prehospital low‐titre group O RhD negative whole blood (LTOWB) as an option for critically bleeding patients. The use and storage of blood differs between prehospital centres and the West Region of Sweden has chosen to always have whole blood available on board the helicopter during all missions. Although monitoring of blood during storage is part of a well‐controlled protocol,^11^, ^12^ there are few studies on how the blood is affected during transport in a helicopter,^13^ even though the environment and handling differ greatly from those at a blood bank. The helicopter operates at different altitudes and temperatures and at the same time adds a significant amount of movement and vibration to the blood products, thereby introducing several risks for altering the coagulation capacity as well as platelet function.

Therefore, our aim of the present study was to evaluate the specific effect of whole blood storage in the helicopter emergency medical service (HEMS), investigating coagulation capacity, platelet function and a conventional blood gas over time. Furthermore, we assessed whether whole blood cold storage on board a helicopter meet the requirement of the European standard,^14^ which are set up for storing, handling and transfusion of blood products.

## 2 METHOD

This was a prospective, single‐centre observational study investigating changes in coagulation and platelet function during storage and use of LTOWB in a physician‐staffed HEMS unit in VGR, Sweden. The helicopter in use is an AgustaWestland AW169 (Leonardo S.p.A., Rome, Italy).

### 2.1 Ethics approval

All procedures were done in accordance with the Declaration of Helsinki and approved by the Swedish Ethical Review Authority (reg. No. 2020‐02274).

### 2.2 Description of European standard for handling of blood products

The units were collected, prepared and stored according to the standard.^14^ The units have been stored in a temperature interval between +1 and 10°C and if a unit surpasses +10°C, its shelf life is shortened, while +14°C leads to it being discarded. The maximum threshold for haemolysis is <0.8%.

### 2.3 Blood collection and preparation

During the study period (February–October 2022), in cooperation with Transfusion Medicine in VGR, LTOWB units were collected from healthy male and female volunteers with blood type O RhD negative, low‐titre anti‐A and anti‐B (immunoglobulin (Ig)G < 512, IgM < 256). The units were screened and confirmed negative for human leukocyte antigen (HLA) class I/II antibodies, and were phenotyped as C‐, D‐, E‐ and K‐negative.

The blood was subsequently filtered through a platelet‐sparing leukocyte depletion filter (IMUFLEX®; Terumo Blood and Cell Technologies, Lakewood, CO, USA) and stored at +2–6°C.

**FIGURE 1:**
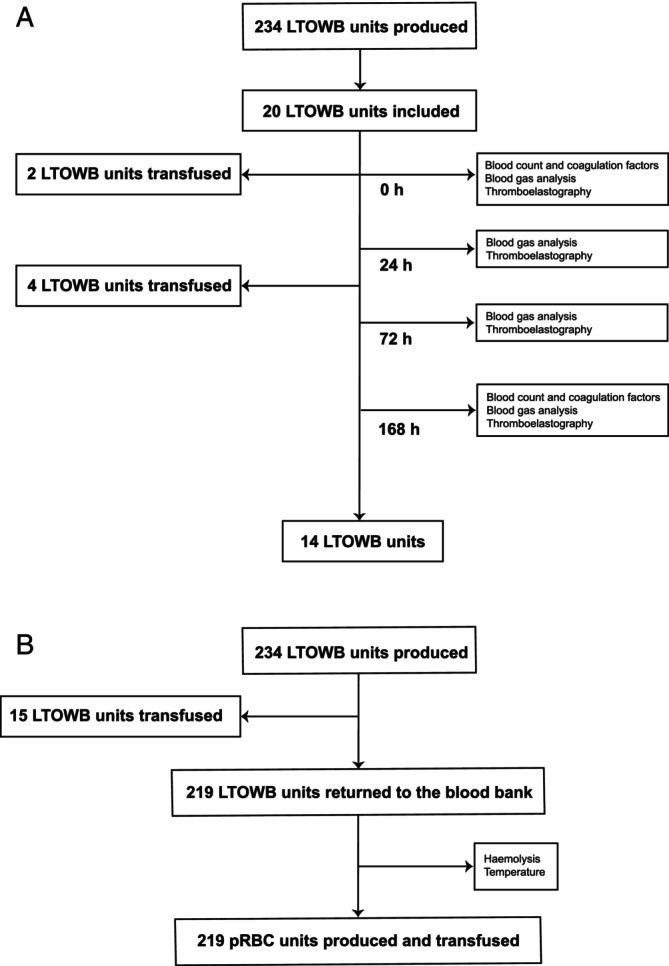
STROBE diagram of tested units flow during the study period. (A) Flow of study units during the study period, with test points, transfused prehospital units and total amount of units with all study points measured. (B) Flow of all units during the study period, with produced units, transfused prehospital units and units returned to the blood bank, tested and separated into packed red blood cells (pRBCs). LTOWB, low‐titre group O RhD negative whole blood; pRBC, packed red blood cells.

A total of six units were prepared each week; four units were sent to the VGR HEMS base and two were stored at the blood bank as a backup for when the sent units were used during the week. All blood products were stored in a temperature‐controlled refrigerator (BioCompact II 210, Gram Scientific ApS, Vojens, Denmark) at +2–6°C, remotely controlled by the blood bank. The units, if unused, were returned to the blood bank after 7 days and prepared as packed red blood cells (pRBCs) for use by the hospital's regular blood bank (Figure 1).

The distributed units were divided in two airtight and approved cooling boxes (Crēdo Cube™; Peli Biothermal EMEA, Leighton Buzzard, UK) with a temperature monitoring unit (Boomerang, ICU Scandinavia AB, Täby, Sweden).

Two units were kept in the helicopter and rotated with the units in the refrigerator every 12 h. Two units were present in the helicopter at all times to ensure that LTOWB was readily available for a mission involving critical bleeding.^11^ If a unit was transfused to a patient, a blood warmer (°M Warmer, °MEQU A/S, Copenhagen, Denmark) was used to avoid hypothermia.

The test units were included in the study from the inventory at the VGR HEMS base; units that were transfused to a patient were lost to follow‐up (Figure 1).

### 2.4 Sampling and analysis

All units were tested upon collection at the blood bank and at the VGR HEMS base (Table S1).

Measurements were done at four time points, at 0, 24, 72 and 168 h. Baseline values were collected at 0 h as standard laboratory analyses for haemoglobin (Hb), platelet count (ADVIA® 2120i Hematology System, Siemens Healthcare AB, Solna, Sweden), prothrombin time/international normalised ratio (PT‐INR), activated partial thromboplastin time (aPTT), antithrombin (thrombin/fXa), fibrinogen, and factors VIII, X and XIII (CS‐2500 and CS‐5100, Sysmex Nordic Aps, Landskrona, Sweden) at the blood bank (Figure 1).

After distribution to the VGR HEMS base, the units were sampled, warmed and tested using thromboelastography (TEG)^15^, ^16^, ^17^ (TEG 6S; Nordic Biolabs, Täby, Sweden), where the assays were done in a multi‐channel cartridge. A drop of blood was added and the clot formation and lysis were displayed as a graph.

To ensure that the blood sampling at the VGR HEMS base did not affect the analysis of the blood, comparative tests were conducted without warming using citrated sampling tubes, according to routine procedure. Since both hypothermia and the added sodium citrate in the sampling tubes negatively affected coagulation, the analysis was made on pre‐warmed samples and collected in non‐citrated sampling tubes in collaboration with the manufacturer and the Department of Haematology and Coagulation Disorders, Sahlgrenska University Hospital, Gothenburg, Sweden.

The following TEG assays were conducted: rapid TEG™ (rTEG), where tissue factor (TF) is added to the kaolin activation to accelerate the assay; TEG functional fibrinogen (citrated functional fibrinogen [CFF]), where TF is added for activation and anti‐glycoprotein (Gp)IIb/IIIa is added for platelet inactivation; and TEG platelet mapping (PM), which evaluates the contribution of agonist‐induced clot strength with adenosine diphosphate (ADP) and arachidonic acid (AA).

The following measurements were collected: *R* = time (min) elapsed between initiation and a 2 mm amplitude reading; *K* = time (min) elapsed between the split point and the point where fibrin cross‐linking produces a 20 mm amplitude; *α* = the angle (°) formed by the slope of a tangent line from the midpoint between the *R* and *K* time; and MA = maximum amplitude, where clot strength reaches its maximum (mm).

Finally, a venous blood gas (VBG) analysis was performed (Epoc®; Siemens Healthcare AB, Solna, Sweden)^18^ for conventional blood gas analysis and plasma concentrations of sodium, potassium, chloride, glucose and lactate.

Subsequently, TEG and a new VBG analysis were performed at 24, 72 and 168 h. At 168 h, when the LTOWB was returned to the blood bank, a new round of laboratory tests for haematology and coagulation were performed, including controls for haemolysis (ADVIA® 2120i Hematology System, Siemens Healthcare AB, Solna, Sweden) and temperature in accordance to European standard.^14^

### 2.5 Statistical analysis

All analyses were performed using SAS® v9.4 (SAS Institute, Cary, NC, USA). Results are presented as mean, median (min;max), sample size (*n*), standard deviation (SD), variance, 95% confidence intervals (CIs) and *p* value (*p*). For comparisons over time, Fisher's non‐parametric permutation test for paired observations was used for continuous variables. A mixed model repeated measurements analysis with unstructured covariance matrix for normal distributed variables was chosen and the mixed model repeated measurements analysis with unstructured covariance matrix was used to calculate the normally distributed variables' mean differences with 95% CI. Statistically significant changes were defined as *p*‐value <.05 and lack of overlap of CIs.

## 3 RESULTS

### 3.1 Low‐titre group O whole blood units

During the study period, 234 LTOWB units were produced and delivered to the VGR HEMS base, 15 were transfused and 219 units were returned to the blood bank (Figure 1). Altogether 626 missions were performed, with a flight time of 670 h.

A total of 20 units were included and 14 units were analysed at all time points (0, 24, 72 and 168 h). Six of the included units were used for transfusion and lost to follow‐up (Figure 1).

A post hoc power calculation was made and for a mean difference of 10, a standard deviation of 12 and an alpha of 0.05%, a total number of 14 samples resulted in a power of 82.2%.

### 3.2 Blood count and coagulation factors

Haemoglobin, prothrombin time, activated prothrombin time, antithrombin, fibrinogen, factor VIII, factor X and factor XIII did not show any significant changes from baseline to 168 h of storage (Table 1).

**TABLE 1:** Blood count and coagulation factors showing changes over time.

	0 h		168 h		0–168 h		*p*
Hb (g/dL)	12.97 ± 0.73	*n* = 20	12.98 ± 0.83	*n* = 17	0.006 ± 0.21 (−0.10; 0.11)	*n* = 17	1.0
PT‐INR (ratio)	1.16 ± 0.16	*n* = 20	1.23 ± 0.12	*n* = 15	0.05 ± 0.17 (−0.04; 0.14)	*n* = 15	.3
aPTT (s)	37.6 ± 9.4	*n* = 20	39.9 ± 8.3	*n* = 15	1.13 ± 4.9 (−1.58; 3.85)	*n* = 15	.4
Antithrombin–thrombin (kUI/L)	0.84 ± 0.12	*n* = 20	0.82 ± 0.098	*n* = 15	−0.011 ± . (−0.028; 0.005)	*n* = 15	.2
Antithrombin–FXa (kUI/L)	0.86 ± 0.14	*n* = 20	0.84 ± 0.13	*n* = 15	−0.019 ± 0.05 (−0.04; 0.006)	*n* = 15	.08
Fibrinogen (g/L)	2.43 ± 0.5	*n* = 20	2.34 ± 0.55	*n* = 15	−0.01 ± 0.04 (−0.03; 0.01)	*n* = 15	.5
FVIII (kUI/L)	0.58 ± 0.3	*n* = 20	0.46 ± 0.25	*n* = 15	−0.04 ± 0.35 (−0.24; 0.15)	*n* = 15	.7
FX (kUI/L)	0.78 ± 0.16	*n* = 16	0.73 ± 0.13	*n* = 13	−0.04 ± 0.07 (−0.076; 0.003)	*n* = 13	.09
FXIII (kUI/L)	1.16 ± 0.22	*n* = 20	1.19 ± 0.24	*n* = 15	0.04 ± 0.17 (−0.05; 0.14)	*n* = 15	.4
Haemolysis (%)	<0.8%	*n* = 234	<0.8%	*n* = 219			

*Note*: Data are presented as mean ± SD for each blood count and coagulation factor variable. For changes mean ± SD/(95% CI for mean) and for haemolysis, the threshold value is presented.

Abbreviations: aPTT, activated partial thromboplastin time; FVIII, factor VIII; FX = factor X; FXIII, factor XIII; Hb, haemoglobin; PT‐INR, prothrombin time/international normalised ratio.

**FIGURE 2:**
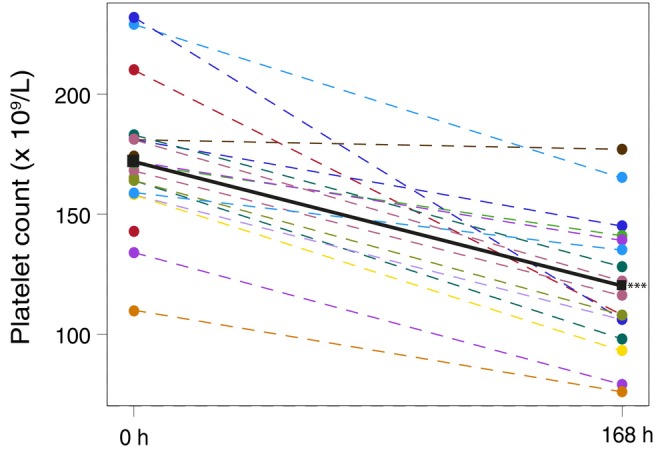
Plot of all time points for the platelet count (×10^9^/L). The filled circles are the individual data for each blood unit connected by a dashed line of the same colour. The filled black square is the mean of all subjects for every time point, connected by a line of the same colour. Platelet count was 172 ± 29 at 0 h and 120 ± 28 at 168 h. Baseline measurements were performed at 0 h. ***p < .0001.

Platelet counts decreased significantly by a mean of 54 × 10^9^/L (95% CI −68; −39) from a baseline mean value of 172 ± 29 × 10^9^/L to a mean value of 120 ± 28 at 168 h (*p* < .0001) (Figure 2).

### 3.3 Electrolytes, glucose, lactate and pH

There was a significant decrease in pH from baseline over time, with a reduction from 7.06 ± 0.04 to 6.91 ± 0.05 (*p* ≤ .0001) at 168 h. The plasma sodium concentration decreased significantly over time from 129.2 ± 2.9 mmol/L at baseline to 121.4 ± 4.7 mmol/L at 168 h (*p* ≤ .0001), while plasma potassium increased over time from 7.3 ± 1.7 to 11.7 ± 0.6 mmol/L (*p* ≤ .0001). Plasma concentration of chloride was not changed at 24 or 72 h but was significantly increased at 168 h (103.7 ± 1.8 mmol/L) compared with baseline (102 ± 1.6 mmol/L) (*p* = .0003). Plasma glucose decreased over time from 20.6 ± 1.3 to 18.0 ± 1.4 mmol/L (*p* = .0001). Plasma lactate increased over time from 5.9 ± 1.2 at baseline to 11 ± 1.4 at 168 h (*p* = .0002) (Table 2).

**TABLE 2:** Blood gas variables showing changes over time.

	0 h	24 h	72 h	168 h	Δ 0–24 (h)	Δ 0–72 (h)	Δ 0–168 (h)
pH (units)	7.06 ± 0.04 *n* = 18	7.04 ± 0.04 *n* = 18	7.0 ± 0.05 *n* = 14	6.91 ± 0.05 *n* = 14	−0.02 (−0.04; −0.0) *n* = 18 ***p* = .05** *	−0.06 (−0.09; −0.03) *n* = 14 ***p* = .0003** ***	−0.16 (−0.17; −0.14) *n* = 14 ***p* ≤ .0001** ***
Na^+^ (mmol/L)	129.2 ± 2.9 *n* = 18	128.8 ± 3.3 *n* = 18	125.0 ± 4.9 *n* = 14	121.4 ± 4.7 *n* = 14	−0.39 (−1.34; 0.56) *n* = 18 *p* = .4	−3.97 (−6.34; −1.59) *n* = 14 ***p* = .003** **	−7.57 (−9.8; −5.3) *n* = 14 ***p* ≤ .0001** ***
K^+^ (mmol/L)	7.3 ± 1.7 *n* = 18	7.8 ± 1.6 *n* = 18	10.1 ± 1.7 *n* = 14	11.7 ± 0.6 *n* = 14	0.54 (0.30; 0.79) *n* = 18 ***p* = .0002** ***	2.68 (1.91; 3.46) *n* = 14 ***p* ≤ .0001** ***	4.42 (3.64; 5.20) *n* = 14 ***p* ≤ .0001** ***
Cl^−^ (mmol/L)	102.0 ± 1.6 *n* = 18	102.1 ± 2.1 *n* = 18	102.5 ± 1.8 *n* = 14	103.7 ± 1.8 *n* = 14	0.056 (−0.98; 1.09) *n* = 18 *p* = .9	0.51 (−0.39; 1.41) *n* = 14 *p* = .3	1.73 (0.93; 2.53) *n* = 14 ***p* = .0003** ***
Glucose (mmol/L)	20.6 ± 1.3 *n* = 18	20.0 ± 1.3 *n* = 18	19.4 ± 1.3 *n* = 14	18.0 ± 1.4 *n* = 14	−0.6 (−0.8; −0.3) *n* = 18 ***p* = .0002** ***	−1.5 (−2.1; −0.8) *n* = 14 ***p* = .0004** ***	−2.9 (−3.5; −2.2) *n* = 14 ***p* = .0001** ***
Lactate (mmol/L)	5.9 ± 1.2 *n* = 17	6.5 ± 1.1 *n* = 18	8.1 ± 1.3 *n* = 14	11.0 ± 1.4 *n* = 14	0.47 (0.2; 0.7) *n* = 17 ***p* = .0007** ***	2.2 (1.6; 2.8) *n* = 13 ***p* = .0002** ***	5.1 (4.5; 5.7) *n* = 13 ***p* = .0002** ***

*Note*: Data are presented as mean ± SD/*n* = for all blood gas variables. For changes mean difference (95% CI for mean)/*n*/*p* is presented. Significant *p* values are highlighted in bold.

Abbreviations: Cl^−^, chloride; K^+^, potassium; Na^+^, sodium.

* *p* < .05;

** *p* < .01;

*** *p* < .001.

### 3.4 Changes in coagulation measured by thromboelastography

The rTEG R value was not significantly changed at any of the measurement time points. The K value increased from 2.1 ± 0.6 min at baseline to 2.9 ± 0.8 min at 168 h (*p* ≤ .0001) and the α angle decreased from 68.9 ± 6.2° at baseline to 65.8 ± 7.8° at 168 h (*p* = .0002). The MA value decreased from 55.8 ± 4.6 mm at baseline to 52.4 ± 5.5 mm at 168 h (*p* = .0006). At 24 or 72 h, *K*, *α* and MA were not significantly changed from baseline (Table 3).

**TABLE 3:** Selected thromboelastography (TEG) variables from the Citrated rapid TEG (CRT), TEG citrated functional fibrinogen (CFF) and Platelet mapping (PM) assays.

	0 h	24 h	72 h	168 h	Δ 0–24 h	Δ 0–72 h	Δ 0–168 h
Citrated rapid TEG (CRT)							
R (min)	0.6 ± 0.2 *n* = 18	0.6 ± 0.2 *n* = 18	0.7 ± 0.2 *n* = 14	0.7 ± 0.3 *n* = 14	−0.006 (−0.1; 0.1) *n* = 18 *p* = 1.0	0.02 (−0.08; 0.1) *n* = 14 *p* = .8	0.08 (−0.08; 0.2) *n* = 14 *p* = .4
K (min)	2.1 ± 0.6 *n* = 18	2.1 ± 0.6 *n* = 18	2.2 ± 0.6 *n* = 14	2.9 ± 0.8 *n* = 14	−0.05 (−0.15; 0.05) *n* = 18 *p* = .3	0.06 (−0.12; 0.25) *n* = 14 *p* = .5	0.73 (0.57; 0.9) *n* = 14 ***p* ≤ .0001** ***
⍺ (°)	68.9 ± 6.2 *n* = 18	69.1 ± 6.1 *n* = 18	68.7 ± 6.1 *n* = 14	65.8 ± 7.8 *n* = 14	0.1 (−0.4; 0.6) *n* = 18 *p* = .6	−0.3 (−1.7; 1.1) *n* = 14 *p* = .8	−3.3 (−4.4; −2.2) *n* = 14 ***p* = .0002** ***
MA (mm)	55.8 ± 4.6 *n* = 18	56.1 ± 4.7 *n* = 18	55.0 ± 4.9 *n* = 14	52.4 ± 5.5 *n* = 14	0.3 (−0.3; 0.8) *n* = 18 *p* = .4	−0.4 (−1.6; 0.9) *n* = 14 *p* = .5	−3.1 (−4.4; −1.7) *n* = 14 ***p* = .0006** ***
TEG functional fibrinogen (CFF)							
MA (mm)	20.3 ± 7.0 *n* = 18	21.5 ± 7.1 *n* = 18	19.5 ± 2.4 *n* = 14	17.6 ± 2.9 *n* = 14	1.2 (0.4; 2.0) *n* = 18 ***p* = .004** **	−1.4 (−6.2; 3.4) *n* = 14 *p* = .9	−3.3 (−7.6; 1.0) *n* = 14 ***p* = .002** **
Platelet mapping							
ADP MA (mm)	58.8 ± 4.0 *n* = 17	59.4 ± 3.5 *n* = 18	57.4 ± 4.0 *n* = 14	54.2 ± 4.6 *n* = 14	0.38 (−0.4; 1.1) *n* = 17 *p* = .3	−1.0 (−2.4; 0.4) *n* = 14 *p* = 0.2	−4.2 (−5.7; −2.7) *n* = 14 ***p* = .0004** ***
AA MA (mm)	58.2 ± 4.4 *n* = 17	58.8 ± 3.9 *n* = 18	56.8 ± 4.2 *n* = 14	52.8 ± 4.7 *n* = 14	0.3 (−0.7; 1.3) *n* = 17 *p* = .5	−1.1 (−2.4; 0.3) *n* = 14 *p* = .1	−5.0 (−6.8; −3.3) *n* = 14 ***p* = .0004** ***

*Note*: Data are presented as mean ± SD/*n* = is presented for each TEG variable. For changes mean difference (95% CI for mean)/*n* = /*p* is presented. Significant *p* values are highlighted in bold.

Abbreviations: ⍺, angle (°); AA, arachidonic acid; ADP, adenosine diphosphate; CFF, citrated functional fibrinogen; CRT, citrated rapid TEG; PM, platelet mapping; K, time elapsed (min); MA, maximum amplitude (mm); R, time elapsed (min).

** *p* < .01;

*** *p* < .001.

### 3.5 Thromboelastrography functional fibrinogen

There were no significant changes in the MA of the functional fibrinogen assay between 0 and 72 h. However, the MA initially increased from 20.3 ± 7.0 to 21.5 ± 7.1 during the first 24 h and then decreased to 17.6 ± 2.9 at 168 h (Table 3). All values on TEG functional fibrinogen were within the reference range provided by the manufacturer (10–25 mm).

### 3.6 Assessing platelet function measured using platelet‐mapping thromboelastography

Platelet mapping for ADP MA and AA MA did not show any significant changes during the first 72 h of storage. After 168 h of storage, however, both values decreased significantly from a mean of 58.8 ± 4.0 mm to 54.2 ± 4.6 mm and from a mean of 58.2 ± 4.4 mm to 52.8 ± 4.7 mm for ADP MA and AA MA, respectively (Table 3). All values at all time points were within the reference range provided by the manufacturer for ADP MA (45–69 mm).

### 3.7 Quality requirements for low‐titre group O whole‐blood units

None of the returned units (219) were discarded and all of the units met the European standard^14^ of storage temperature limits (+1–10°C) and haemolysis (<0.8%). All separated pRBC units were used in patients without any reported adverse reactions.

## 4 DISCUSSION

This prospective observational study examined coagulation capacity and platelet function over time in whole blood (LTOWB) during helicopter missions of a physician‐staffed HEMS unit. The main findings were that the coagulation factors' capacity was well preserved for 168 h and that coagulation and platelet function, as measured by TEG, were slightly affected. Furthermore, the handling of LTOWB during emergency missions complied with the European standard.^14^

It has been reported that, during the wars in Afghanistan and Iraq, most of the preventable deaths were due to massive haemorrhage.^19^ Having the ability to give early^20^, ^21^, ^22^ transfusion in major haemorrhage is of paramount importance and an essential part of damage control resuscitation (DCR).^23^, ^24^ The ability to give a transfusion of whole blood instead of the separated and diluted^25^, ^26^ components may hold several advantages, as discussed by Brill et al.,^27^ besides the logistical one of enabling handling and transport in a helicopter.

Recent, prospective observational studies have shown that whole blood is associated with reduced mortality compared with blood component therapy.^28^, ^29^ It is therefore likely that the use of whole blood would be the method of choice for prehospital resuscitation of major haemorrhage. In this respect, it is therefore important that the quality of LTOWB units during transport and handling is investigated when used in an HEMS setting.

The platelet count decreased by 30% over a 7‐day period, which corresponds to a decline of approximately 4% per day during the study period. In a recent review by Van der Meer et al.,^30^ it was shown that platelet count decreased by 1%–2% per day on average in cold‐stored whole blood, while another study showed a decline of almost 50% over 7 days.^31^ Refrigeration of platelet concentrates causes morphological changes, leading to a clearance from the circulation in 1–2 days post‐transfusion via macrophages,^32^ although in vitro and in vivo^33^, ^34^ studies show better or maintained coagulation properties, at least with a storage limit of 14 days.^35^

Despite the fall in platelet count, platelet function was only moderately affected, having decreased by 8%–9% after 168 h of handling and transport. The effect on platelet count and preserved coagulation function, as measured by TEG, in our samples matches the data from McRae et al.,^36^ who monitored cold‐stored LTOWB units for 28 days. A degradation of platelet function in cold‐stored whole blood as early as 7 days in hospital has been reported by Assen et al.,^35^ although with preserved clot formation. Previous studies suggest that cold‐stored platelets have better haemostatic properties,^34^ but there are also conflicting data where the filtration of whole blood resulted in decreased clotting capacity and thrombin generation,^37^ but increased levels of anaphylatoxins,^38^ and where the platelets were deemed unsuitable for haemostatic resuscitation. When performing massive transfusions with LTOWB, we therefore suggest awareness of low platelet count and function.

In the present study, the storage and handling of LTOWB at our HEMS unit did not affect the levels of fibrinogen and factors VIII, X and XIII. This is in contrast to a previous study, by Nilsson et al.,^39^ who showed that, at 7 days, coagulation factor VIII decreased to 58% of original activity, while factors XII, IX, V and XIII, plasminogen and fibrinogen remained at stable levels in cold‐stored whole blood. Decreased function of factor VIII over 14 days was shown by Susila et al.,^25^ who also reported, in contrast to our study, a significant increase in aPTT. Bjerkvig et al.,^10^ also noted this difference between the units stored at the blood laboratory and the units stored at a HEMS base, but only after 21 days.

Regarding metabolic variables in our LTOWB units, we found a decrease in pH, glucose and sodium. An increase over time was seen in both lactate and potassium. Our results are consistent with, though in some cases less pronounced than, previous findings by Bjerkvig et al.,^10^ and Pulliam et al.^40^ The changes are possibly attributable to the anaerobic properties of the thermal box in which the blood units are contained, as suggested by Bjerkvig et al.^10^ Compared with the blood components that are transfused in a hospital setting, they show less acidity and lower potassium levels.^41^ There needs to be awareness of the potential risk of low pH and high lactate and potassium levels in whole blood transfusion.

Regarding TEG, our results show similar properties to previous findings over 14 days as reported by Susila et al.,^25^ 21 days as reported by Bjerkvig et al.,^10^ and 31 days as reported by Jobes et al.,^42^ although some of these studies were performed with rotational thromboelastometry (ROTEM®) instead of TEG.^25^ Our findings of preserved levels of functional fibrinogen, as measured by TEG, are consistent with previous findings.

The study by Bjerkvig et al.,^10^ on properties of cold‐stored LTOWB was done in HEMS units where the blood units were brought on board only if the alarm operator informed the HEMS crew of a potential haemorrhagic trauma. By contrast, in our setting, we routinely bring LTOWB units on all flights, irrespective of the nature of the trauma. We found preserved coagulation properties, and slightly reduced platelet capacity, matching the results of the studies storing the blood products at the base.

Prehospital handling leads to many concerns as the handling chain is not as controlled as in a hospital with trained technicians, although a systematic review^43^ challenged the adverse effect of temperature variation on the efficiency of blood products.

None of the LTOWB units were discarded during the study period after testing for haemolysis and temperature and the subsequent pRBCs were all transfused to patients. Our conclusion is that the handling and storage on board the helicopter holds up to European standard.^14^ We therefore suggest that whole blood can be brought on board on all helicopter missions, which has, to our knowledge, not previously been shown.

Our study has limitations. The units lost to transfusion were a calculated limitation as we wanted to use the LTOWB units that were in clinical use to mimic the handling of the units. More sensitive and exclusive measures of coagulation, for example, PTF and TAT, were not undertaken in this study. Another limitation is that the number of units required for drawing confident conclusions is hard to calculate, as only a very limited number of studies have been performed on this subject.

## 5 CONCLUSIONS

Storage and transport of whole‐blood units in a helicopter with many flight hours and long flight times had some influence on blood quality, in terms of coagulation capacity and platelet number and function, up to 168 h. When performing transfusions with whole blood there should be an awareness of low platelet count, low pH and high levels of potassium and lactate.

In this study, we demonstrated that handling and storage of whole blood on board of the helicopter holds up to European standard,^14^ measured as temperature and haemolysis.

Further studies are needed to evaluate if these changes have an effect on patient outcome.

## AUTHOR CONTRIBUTIONS

JS, BR, AW and SR designed the study. JS performed all the experiments. AW and VR contributed to sample preparations and analysis. JS took the lead in writing the manuscript under supervision from BR. All authors provided critical feedback and helped shape the research, analysis and manuscript.

## FUNDING INFORMATION

The Helicopter Emergency Medical Service, Region Västra Götaland supported funding for the blood products, laboratory equipment and laboratory analysis. The Swedish Armed Forces Science & Technology Fund (grant no. AT9221011) provided funding for a TEG® 6S system. The Swedish Society for Military Medical Officers supported funding for a Siemens Epoc® blood gas analyser and laboratory equipment. The Local Research and Development Council Fyrbodal supported funding for writing of the study and ethical application development. The Centre for Disaster Medicine, Gothenburg University, supported funding for writing of this article and for statistical analysis. The Department of Haematology and Coagulation Disorders at Sahlgrenska, Gothenburg, supported funding for the laboratory analysis.

## CONFLICT OF INTEREST STATEMENT

The authors declare that they have no conflicts of interest.

## Supporting information


**Data S1.** Supporting information.

## Data Availability

The data that support the findings of this study are available from the corresponding author upon reasonable request.
