# Supplementary material for: Coagulation and platelet function in cold‐stored whole blood on missions in a helicopter emergency service
Source: Acta Anaesthesiol Scand. 2025 Jan 19;69(2):e14568. doi: 10.1111/aas.14568 (PMC11744058; doi:10.1111/aas.14568)
Supplement: Supplementary file 1 — Data S1. Supporting information. [file AAS-69-0-s001.pdf]

Supplemental data

Supplemental table 1

Overview of samples and time points.

Analysis	0 hrs	24 hrs	72 hrs	168 hrs
**Laboratory**	Hb (g/dL)<br>Haemolysis (%)<br>Platelet count (10^9/L)<br>PT-INR (ratio)<br>aPTT (s)<br>HCt (%)<br>Antithrombin (kIU/L)<br>Fibrinogen (g/L)<br>FVIII, FX, FXIII (kIU/L)			Hb (g/dL)<br>Haemolysis (%)<br>Platelet count (10^9/L)<br>PT-INR (ratio)<br>aPTT (s)<br>HCt (%)<br>Antithrombin (kIU/L)<br>Fibrinogen (g/L)<br>FVIII, FX, FXIII (kIU/L)
**Rapid TEG™ (CRT)**	R (min)<br>K (min)<br>α (°)<br>MA (mm)	R (min)<br>K (min)<br>α (°)<br>MA (mm)	R (min)<br>K (min)<br>α (°)<br>MA (mm)	R (min)<br>K (min)<br>α (°)<br>MA (mm)
**TEG functional fibrinogen (CFF)**	MA (mm)	MA (mm)	MA (mm)	MA (mm)
**TEG Platelet mapping (PM)**	ADP MA (mm)<br>AA MA (mm)	ADP MA (mm)<br>AA MA (mm)	ADP MA (mm)<br>AA MA (mm)	ADP MA (mm)<br>AA MA (mm)
**Blood gas**	pH (units)<br>Na^+ (mmol/L)<br>K^+ (mmol/L)<br>Cl^- (mmol/L)<br>HCt (%)<br>Hb (g/dL)<br>Glucose (mmol/L)<br>Lactate (mmol/L)	pH (units)<br>Na^+ (mmol/L)<br>K^+ (mmol/L)<br>Cl^- (mmol/L)<br>HCt (%)<br>Hb (g/dL)<br>Glucose (mmol/L)<br>Lactate (mmol/L)	pH (units)<br>Na^+ (mmol/L)<br>K^+ (mmol/L)<br>Cl^- (mmol/L)<br>HCt (%)<br>Hb (g/dL)<br>Glucose (mmol/L)<br>Lactate (mmol/L)	pH (units)<br>Na^+ (mmol/L)<br>K^+ (mmol/L)<br>Cl^- (mmol/L)<br>HCt (%)<br>Hb (g/dL)<br>Glucose (mmol/L)<br>Lactate (mmol/L)

Laboratory blood samples: aPTT = activated partial thromboplastin time; antithrombin = thrombin and fXa-based method; fibrinogen; factors VIII, X and XIII; haemolysis = presence of haemolysis; Hb = haemoglobin; platelet count; PT-INR = prothrombin time/international normalized ratio.

TEG values: AA = arachidonic acid; ADP = adenosine diphosphate; CFF = citrated functional fibrinogen; CRT = citrated rapid TEG; PM = platelet mapping. α = angle (°); K = time elapsed (min); MA = maximum amplitude (mm); R = time elapsed (min).

Blood gas values: Cl^- = chloride; HCt = haematocrit; Hb = haemoglobin; glucose; K^+ = potassium; lactate; Na^+ = sodium; pH
